# Artificial neural network and response surface methodology for optimization of corrosion inhibition of mild steel in 1 M HCl by *Musa paradisiaca* peel extract

**DOI:** 10.1016/j.heliyon.2022.e11955

**Published:** 2022-11-29

**Authors:** Olusola S. Amodu, Moradeyo O. Odunlami, Joseph T. Akintola, Tunde V. Ojumu, Olushola S. Ayanda

**Affiliations:** a Department of Chemical Engineering, Lagos State University of Science and Technology, Ikorodu, Lagos, Nigeria; b Department of Chemical Engineering, University of Lagos, Akoka, Lagos, Nigeria; c Department of Chemical Engineering, Cape Peninsula University of Technology, Cape Town, South Africa; d Department of Chemistry, Federal University of Oye-Ekiti, Nigeria

**Keywords:** Artificial neural network, Banana peel extract, Corrosion, Inhibition, Response surface methodology, Optimization

## Abstract

Banana (*Musa paradisiaca*) peel extract (BPE) was used as an environmentally benign corrosion inhibitor for mild steel in 1.0 M HCl. The efficiency of BPE was assessed by monitoring the pH of HCl solution and the quantity of hydrogen gas evolved during the reaction, using gasometric and thermometric methods. Moreover, the effect of concentration and temperature on the inhibition efficiency was modelled and optimized by response surface methodology (RSM) and artificial neural network (ANN). It was observed that the evolution of hydrogen gas decreases with increasing concentration of BPE, while it increases with time for the various concentrations up till 8 min before attaining constant values. By numerical optimization of RSM, the optimum corrosion inhibition efficiency of 60.08% was obtained at 308.08 K and concentration of 7.44 g/L for gasometric method, while an optimum of 61.25% was obtained at 308 K and 7.50 g/L for thermometric method. Optimization of inhibition parameters with ANN revealed the optimum number of neurons for both gasometric and thermometric methods to be 7; while the MSE are 2.2788 and 2.7306, and R^2^ are 96.21 and 98.86 respectively. Comparing the performance models of RSM and ANN: for gasometric method, R^2^ was 98.93 for RSM and 96.21 for ANN, while for thermometric method, R^2^ was obtained as 95.78 and 98.86 for RSM and ANN, respectively. Both RSM and ANN have demonstrated to be robust optimization techniques; particularly, ANN was found to give a good prediction of the available small dataset.

## 1 Introduction

The rising cases of corrosion problems in pipelines, refineries, water treatment and petrochemical plants, as well as in power generation (nuclear, thermal, hydropower etc.) plants in recent time have been attributed to industrialization [1]. However, deployment of suitable corrosion prevention and/or inhibition methods can significantly circumvent this challenge. Basically, corrosion is the tendency for metals to deteriorate to their natural ore state by an electrochemical process in the presence of moisture and oxygen, which makes the bulk metal to break up thereby losing its useful properties. This process takes place when two unlike metals in contact are dipped in the same corrodent solution, or when same metal is immersed in an electrolyte with different concentrations, in which case the anodic metal becomes corroded while the cathode is protected [2], [3], [4]. The conversion of metal to the low-grade ore during this reaction is accelerated by the movement of electrons on the corroded cells; this is known as galvanic cell.

Although corrosion is a natural phenomenon, it can be assessed, controlled and prevented by using suitable techniques. Among the methods available for evaluation of corrosion rates, electrochemical impedance spectroscopy, weight loss technique, and hydrogen gas evolution are commonly reported [5], [6], [7]. Whereas each of these techniques has one limitation or another, hydrogen gas evolution method seems to be the most reliable in assessing corrosion rates as the mole of metal dissolved correlates proportionally to the volume of hydrogen gas evolved. Other than water and oxygen, factors that affect metal corrosion rates include pH, salinity, temperature, types of metal involved and inhibitors [8], [9], [10].

The applications of some corrosion inhibitors, particularly the inorganic-based inhibitors, have been restricted due to the growing concern about their toxicity to the environment [11], [12]. In 2019, the global market for corrosion inhibitors was estimated at USD 7.4 billion, with a projection of 3.8% annual growth rate from 2020 to 2027 [1]. What can be adduced for this market growth is the global and gradual awareness, and development of innocuous bio-based corrosion inhibitors. Besides environmental consideration in favour of bio-based corrosion inhibitors, the presence of readily available, effective and economic organic precursors could sustain their market growth. Interestingly, a plethora of agrowastes and plant extracts such as Cannabis plant, *Argemone Mexicana*, *Carica papaya* leaves, tobacco, kolanut, *Musa paradisiaca*, *Coffea Arabica*, *Ferula hermonis*, *Rosmarinus Officinalis*, etc., have been reported as suitable corrosion inhibitors [13], [14], [15], [16], [17], [18].

Generally, corrosion inhibitors contain multiple bonds in their molecules in addition to hetero-atoms such as oxygen, nitrogen etc., by which they adsorb to metal surfaces [19], [20]. The strength of adsorption of inhibitor molecules to metal surface is determined by the functional groups, molecular structure of the molecule, electron density and *π*-electrons. Inhibitors function basically by forming a blanket on metal surface, thus protecting it from corrodent solutions. It has also been reported that the main constituents of plant extracts that enhance their effectiveness as metal inhibitors include gallic acid, flavonoids, steroids, tannic acid, sugars, and alkaloids, which have been identified in the analysis of *Musa paradisiaca peel extracts* [21], [22], [23]. In addition, the efficiency of corrosion inhibitors, influenced by certain parameters such as – concentration of inhibitor, reaction temperature, pH of corrodent etc., can be optimized using suitable optimization tools.

In recent time, more findings have reported on modelling and optimization of metal corrosion inhibition using response surface methodology (RSM) and artificial neural network (ANN). For instance, both RSM and ANN have been applied to model and optimize the inhibition of mild steel in sulphuric acid [24], inhibitive action of *Ocimum basilicum* essential oil as green corrosion inhibitor for C38 steel in 0.5 M H_2_SO_4_ [25] and the performance of surfactant as a corrosion inhibitor for mild steel in 1 M H_2_SO_4_ [26]. Moreover, RSM and ANN have been used effectively in the optimization studies of extraction of oil [27], [28], turbidity removal from produced water [29], and adsorption of heavy metals from wastewater [30]. RSM is a robust tool because it offers a lot of statistical tools to assess the level of interactions of independent variables that eventually gives the highest process efficiency. Some of these tools are: Box-Behnken designs (BBD) and central composite design (CCD), which specify the minimum number of experimental runs; response surface graphs, used to assess the level of interactions among variables; analysis of variance (ANOVA), which tests model fitness to experimental data; mathematical models and numerical optimizations tools. Thus, RSM is significant for modeling and optimization of processes, where the response or dependent variable of interest is influenced by various variables [31], [32]. Similarly, artificial neural network (ANN) has found applications in quantifying nonlinear relationship existing between a model response and independent variables by iterative training of dataset obtained. The network can be adapted for machine learning and pattern recognition to compute response from inputted values via the learning process by adjusting the synaptic connection existing between the neurons [30], [33], [34]. Hence, it simulates specific information analogous to the operation of the human biological neural network [35].

Although ANN has been found to be promising with large dataset, application for available experimental data could identify its predicting strength with smaller dataset. Conversely, RSM has a specific advantage when reduction of experimental runs and highly predictive models are desirable. The suitability of Banana (*Musa paradisiaca*) peel extract (BPE) to inhibit mild steel corrosion in 1 M HCl has been shown in our previous study [18]. The aim of this present work, therefore, was to model and optimize the efficiency of BPE as mild steel corrosion inhibitor in acidic medium using RSM and ANN algorithm. The interactive effect of temperature and inhibitor concentration was investigated, with a view to developing a mathematical model that may be used to predict the correlation between the system response and the input parameters. Finally, the system was further improved by numerical optimization.

## 2 Materials and methods

### 2.1 Samples and reagents

The coupons used in this study were obtained by mechanically press-cutting of mild steel sheet into coupons of dimensions 4 x 2.5 x 0.1 cm, having the following compositions (% wt): Fe – 99.3, Ni – 0.043, Mn – 0.34, Al – 0.03, Cu – 0.069, Co – 0.069 and Ca – 0.087 [18]. Banana peels (ripe), obtained as agro- or domestic waste, were washed under running water and air dried until a constant mass was recorded. Banana peel extract (BPE) is composed of crude fat (3.8-11%), total dietary fibre (43.2-49.7%), crude protein (6-9%), starch (3%), micronutrients (Mg, K, Ca, P), pectin, amino acid and polyunsaturated fatty acids [22]. Moreover, the BPE possesses the following physical properties: Density (1.56 g L^−1^ cm^−3^), viscosity (32.04 cp), surface tension (18.0 dynes cm^−1^), specific gravity (1.55) and flash point (273 °C) [18]. Hydrochloric acid (HCl), acetone and ethanol were obtained from Sigma Aldrich (Sigma-Aldrich, Inc., USA). All reagents and chemicals used were of analytical grade (purity >98%).

### 2.2 Corrosion test solutions preparation

Ethanol was applied to degrease the coupons, while acetone was used to dry them before being transferred into a moisture free desiccator until use. The dried peels were ground into fine powder (about 0.25 mm) and extracted using 95% ethanol. A stock solution of the powered peels was prepared by dissolving 5 g powder in 200 mL ethanol; thereafter, triple filtered while excess ethanol was removed using rotatory evaporator. Corrosion test solution was obtained in the concentration ratios of 1.46, 2.5, 5.0, 7.5 and 8.54% (v/v) by diluting the filtrate (inhibitor) with the corroding agent (i.e., 1 M HCl in distilled water). The procedure was repeated for the control experiments but without adding inhibitor.

### 2.3 Corrosion inhibition efficiency of banana peel extract (BPE)

#### 2.3.1 Gasometric method

The number of experiment conducted was designed and generated by a MINITAB 17.0 software (Stat-Ease Inc., USA). The variables studied were analyzed, each at five coded levels as -*α*, -1, 0, +1, and +*α*, representing negative outlier, low value, centre point, high value and positive outlier, respectively (Table 1), giving a total number of 13 experimental runs.

**Table 1:** Experimental design.

Levels	-*α*	-1	0	+1	+*α*
Concentration (g/L), X_1_	1.46	2.5	5.0	7.5	8.54
Temperature (K), X_2_	306.96	308.0	310.5	313.0	314.04

The coupons were immersed for 6 h in the prepared test solutions at 306.96 K, thereafter withdrawn and washed in detergent solution, rinsed with distilled water, and air-dried. This was repeated for temperatures - 308 K, 310.5 K, 313 K and 314.04 K. The quantity of hydrogen gas discharged in the presence of inhibitor, $V_{1}$, and in the absence of inhibitor, $V_{0}$, were determined. The volume of gas evolved was correlated to corrosion rate by gasometric method. Likewise, corrosion inhibition efficiency (*ε*) was determined at concentrations of 1.46, 2.5, 5.0, 7.5, and 8.54 g/L from Equation (1), as well as the degree of surface coverage (*θ*) from Equation (2) [18].

(1) $$\varepsilon =\left(1-\frac{v_{1}}{v_{0}}\right)⁎100$$

(2) $$\theta =\left(1-\frac{v_{2}}{v_{0}}\right)$$

#### 2.3.2 Thermometric method

Reaction number, $R_{N}$ (°C/min), was determined as a function of temperature rise per time as reported by Ebenso, et al. [36], using Equation (3):

(3) $$R_{N}=(T_{m}-T_{i})/t$$

where $T_{m}$ is the maximum temperature while $T_{i}$ is the initial temperatures attained by the system and *t* is the time. Similarly, corrosion inhibition efficiency (*ε*) was evaluated by reaction number correlation (Eq. (4)) for concentrations 1.46, 2.5, 5.0, 7.5, and 8.54 g/L, at various temperatures.

(4) $$\varepsilon =\left(\frac{R_{N_{O}}-R_{N_{i}}}{R_{N_{o}}}\right)⁎100$$

where $R_{\mathrm{Ni}}$ and $R_{\mathrm{No}}$ are the reaction number of solution with and without inhibitor, respectively. The results presented were three replicate measurements from two flasks.

### 2.4 Statistical analysis and modeling with RSM

The data obtained from the corrosion study were analyzed statistically by response surface methodology. Based on the regression analysis, the response variable was correlated to the independent variables, thereby generating statistical models to describe experimental data. Furthermore, the fitness of the models was determined by ANOVA by assessing the significance of each variable on the system response. Mathematical models were then generated by a Design Expert 10.0.3 (Stat-Ease Inc., USA) for both gasometric and thermometric methods, which relate the response (*Y*) measured to the independent variables as shown in Equation (5).

(5) $$Y=\alpha_{0}+\sum_{i=1}^{n}\alpha_{i}\chi_{i}+\sum_{i=1}^{n}\alpha_{ii}\chi_{i}^{2}+\sum_{i=1}^{n-1}\sum_{j=i+1}^{n}\alpha_{ij}\chi_{i}\chi_{j}+\varepsilon$$

where $X_{1}$, $X_{2}$, $X_{3}$, …, $X_{n}$ are the independent variables, $\alpha_{0}$ is the offset term, while $\alpha_{i}$, $\alpha_{ii}$, and $\alpha_{ij}$ measure linear, squared, and interaction effects, respectively, and *ε* is the random error. Other statistical equations applied are presented in Equations (6)-(8).

(6) $$R^{2}=1-\frac{\sum_{i=1}^{N}{\left(Y_{i}-{\overset{ˆ}{Y}}_{i}\right)}^{2}}{\sum_{i=1}^{N}{\left(Y_{i}-\bar{Y}\right)}^{2}}$$

where:

$$Y_{i}=\text{observed data or experimental data}$$

$${\overset{ˆ}{Y}}_{i}=\text{calculated or predicted data}$$

$$\bar{Y}=\text{mean value of observed data}$$

(7) $$R_{\mathrm{Adjusted}}^{2}=1-\frac{\left(1-R^{2}\right)\left(N-1\right)}{N-P-1}$$

(8) $$\mathrm{MSE}=\frac{1}{N}\sum_{i=1}^{N}{\left(Y_{i}-{\overset{ˆ}{Y}}_{i}\right)}^{2}$$

where *N* is the number of data point, *P* is the number of independent variables and *MSE* is the Mean Squared Error.

### 2.5 Artificial Neural Network (ANN) – computational algorithm

**Figure 1:**
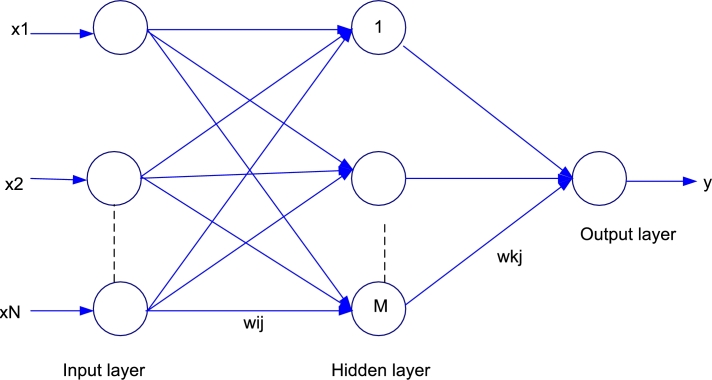
A general Artificial Neural Network (ANN) feed-forward network.

Artificial Neural Network Software (ANN) Toolbox, MATLAB V17 (MathWorks, Inc., USA), was used to model and optimize the efficiency of BPE as mild steel corrosion inhibitor. The inhibition efficiency was predicted using the model generated by Multilayer Normal Feed Forward Neural Network. This network was trained by varying the number of neurons (hidden layers) to get the optimal neurons that can functionally give the best fit for the model, using the value of coefficient of determination (R^2^) obtained at the different number of neurons. Fig. 1 represents the ANN structure of the feed forward network, indicating the input layer (independent variables), hidden layer (number of neurons) and output layer (the response variable or target).

Here, the input layers are the concentration (g/L) and temperature (K); number of neurons are 7, 9, 10, 12, 13, 15, 20, 25 and 30; and the output layer is the inhibition efficiency (%). The network was trained using MSE algorithm at optimum neuron number, while training performance was done by randomly diving the dataset (sample) into three different samples; training (70%), validation (20%) and testing (15%), as earlier performed by Braimah [37].

## 3 Results and discussion

### 3.1 Evaluation of corrosion inhibition efficiency of BPE

**Figure 2:**
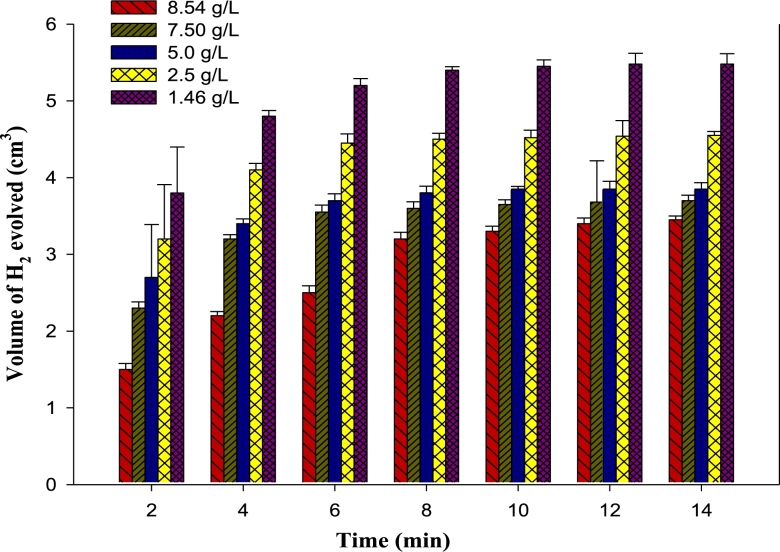
Hydrogen gas evolution rate with varying concentrations of HCl acid solution during corrosion study of mild steel.

The volume of gas evolved from cathodic reaction during the corrosion study (Fig. 2) correlates to corrosion rate as well as inhibition efficiency by gasometric method.

Fig. 2 shows that hydrogen gas evolution of decreases as the concentration of the extract increases, at different immersion times. This is expected since adsorption increases with concentration until equilibrium sorption is attained. On the other hand, the volume of hydrogen gas evolved increases with time for the various concentrations up till 8 min before attaining constant values, which indicates a decrease in adsorption with time. This trend is well reported in metal corrosion inhibition studies [38], [39], [40]. Basically, the dissolution of mild steel in hydrogen chloride generates a molecule of H_2_ and one mole of ferrous ion. Hence, by conservation of mass, an increase in ferrous ions indicates a decrease in mild steel corrosion rate.

**Figure 3:**
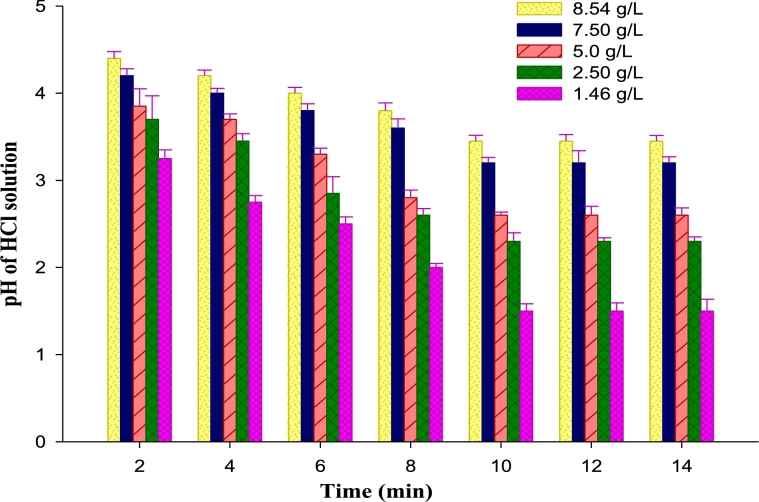
Variation of pH with concentration of HCl solution and immersion time of mild steel in the presence of banana peel extracts (BPE) as inhibitor.

From electrochemical basis of corrosion, the removal and subsequent flow of chloride ion from cathode results in anodic oxidation of iron, thereby releasing electrons to the metal to form hydrated iron (II) chloride (rust). Consequently, to inhibit corrosion, the chloride ion must be prevented from contacting the metal surface. Mild steel corrosion has been found to increase with increased acid concentration [41], [42], [43]. Similarly, Al-Moubaraki, et al. [44] found that corrosion of aluminum increased with increase in concentration of sodium hydroxide, as well as increase in hydrogen gas evolution. Fig. 3 shows that the pH of HCl solution decreases with increased immersion time, due to increased acidity of the solution.

Hydrogen gas evolution rate was used as a measure of corrosion inhibition efficiency at various temperatures and concentrations of BPE. As previously described, the independent variables were analyzed, each at five levels, with a total of 13 experiments conducted. The system response variable which is the efficiency of corrosion inhibition was determined by gasometric and thermometric methods (Table 2).

**Table 2:** Corrosion inhibition efficiency of Banana peel extract – data obtained using gasometric and thermometric methods (experimental results).

Run	A: Concentration (g/L)	B: Temperature (K)	Inhibition (%)	
			Thermometric	Gasometric
1	5.0	310.5	48.31	50.54
2	8.54	310.5	58.66	59.95
3	5.0	310.5	48.31	50.54
4	5.0	306.96	62.56	54.40
5	1.46	310.5	37.96	41.14
6	7.5	308.0	58.39	59.92
7	5.0	310.5	48.31	50.54
8	5.0	310.5	48.31	50.54
9	5.0	314.04	44.07	46.67
10	2.5	313.0	40.31	44.43
11	7.5	313.0	51.73	53.14
12	2.5	308.0	49.25	46.62
13	5.0	310.5	48.31	50.54

From Table 2, comparing runs 5, 1 and 2, there was significant increase in corrosion inhibition efficiency for both methods which was due to changes in concentration from 1.46 to 5.0 g/L, and then to 8.54 g/L since the experimental runs were carried out isothermally. However, for runs 8 and 9, temperature increase from 310.5 K to 314.04 K did not necessarily show corresponding increase in corrosion inhibition efficiency even though the runs were conducted at constant concentration. Similarly, the results for runs 10 and 12 show that temperature elevation does not translate into increased inhibition efficiency. The same trend had earlier been reported for *Musa Paradisiaca* extract on mild steel in sulphuric acid solution [45]. Likewise, the efficiency of *Musa Acuminata* fruit peel extract as corrosion inhibitor for mild steel in acid medium was found to increase with increase in concentration but decreases with increase in temperature [46]. In a corrosion inhibition study using a synthetic inhibitor, and/or synthetic mixed-type inhibitors for mild steel in HCl, the efficiency of the inhibitors reportedly decreased with increasing temperature as well as acid concentration [47]. Akinbulumo, et al. [48] also observed that with *Euphorbia heterophylla* extract as mild steel corrosion inhibitor in 1.5 M HCl, the inhibition efficiency initially increased with temperature for the various immersion time studied but later declined. However, Farhadian, et al. [49] have reported a castor oil-based corrosion inhibitor for mild steel in acidic medium that was able to surmount the challenge of low performance of many inhibitors at high temperature. Overall, the highest efficiency recorded in this study was 62.56% for gasometric method, while 59.95% was obtained for thermometric method, both at low temperature level.

### 3.2 Modeling and statistical analysis

The interaction between the independent variables and their effects on corrosion inhibition efficiency was modelled based on the Regression Fit Model' of MINITAB 17.0 (Pen, USA). Whereas the data obtained using gasometric method was modelled with a response surface 2FI model, quadratic model fit well the experimental data from thermometric technique. The significance of each variable on corrosion inhibition was further assessed by ANOVA (Table 3, Table 4, Table 5, Table 6).

**Table 3:** ANOVA for Response Surface 2FI model (for data obtained using gasometric method).

Source	Sum of Squares	df	Mean Square	F Value	Prob > F	Significance
Model	350.16	3	116.72	278.32	<0.0001	Significant
*A-Concentration (g/L)*	*295.38*	*1*	*295.38*	*704.33*	<*0.0001*	***
*B-Temperature*	*49.51*	*1*	*49.51*	*118.06*	<*0.0001*	***
*AB*	*5.27*	*1*	*5.27*	*12.56*	*0.0063*	***
Residual	3.77	9	0.42			
*Lack of Fit*	*3.77*	*5*	*0.75*			
*Pure Error*	*0.000*	*4*	*0.000*			
Cor Total	353.94	12				


R square information			
Std. Dev.	0.65	R-Squared	0.9893
Mean	50.69	Adj R-Squared	0.9858
C.V. %	1.28	Pred R-Squared	0.9576

The Model F-value of 278.32 implies the model is significant, and there is only a 0.01% chance that a “Model F-Value” this large could occur due to noise. Values of “Prob > F” less than 0.0500 indicate model terms are significant, while values greater than 0.1000 indicate the model terms are not significant. In this case A, B, AB are significant model terms.

**Table 4:** Statistical coefficient value for gasometric method.

Factor	Coefficient Estimate	df	Standard Error	95% CI Low	95% CI High	VIF
Intercept	50.69	1	0.18	50.28	51.10	
A-Concentration (g/L)	6.08	1	0.23	5.56	6.59	1.00
B-Temperature	-2.49	1	0.23	-3.01	-1.97	1.00
AB	-1.15	1	0.32	-1.88	-0.42	1.00

**Table 5:** ANOVA for Response Surface Quadratic model (for data obtained using thermometric method).

Source	Sum of Squares	df	Mean Square	F Value	Prob > F	Significance Level
Model	567.77	5	113.55	31.77	0.0001	Significant
*A-Concentration (g/L)*	*310.43*	*1*	*310.43*	*86.86*	<*0.0001*	***
*B-Temperature*	*217.94*	*1*	*217.94*	*60.98*	*0.0001*	***
*AB*	*1.30*	*1*	*1.30*	*0.36*	*0.5655*	*NS*
$A^{\text{2}}$	*0.35*	*1*	*0.35*	*0.097*	*0.7643*	*NS*
$B^{\text{2}}$	*36.17*	*1*	*36.17*	*10.12*	*0.0155*	***
Residual	25.02	7	3.57			
*Lack of Fit*	*25.02*	*3*	*8.34*			
*Pure Error*	*0.000*	*4*	*0.000*			
Cor Total	592.79	12				


R square information			
Std. Dev.	1.89	R-Squared	0.9578
Mean	49.58	Adj R-Squared	0.9276
C.V. %	3.81	Pred R-Squared	0.6999

The Model F-value of 31.77 implies the model is significant. (***) – significant model terms, while NS – Not significant.

**Table 6:** Statistical coefficient value for thermometric method.

Factor	Coefficient Estimate	df	Standard Error	95% CI Low	95% CI High	VIF
Intercept	48.31	1	0.85	46.31	50.31	
A-Concentration (g/L)	6.23	1	0.67	4.65	7.81	1.00
B-Temperature	-5.22	1	0.67	-6.80	-3.64	1.00
AB	0.57	1	0.95	-1.67	2.81	1.00
A^2^	-0.22	1	0.72	-1.92	1.47	1.02
B^2^	2.28	1	0.72	0.59	3.98	1.02

Based on the Lack-of-Fit Test, the statistical model summary obtained explains the fitness of 2FI models. Empirical models obtained from Equation (5) to predict the efficiency of corrosion inhibition at various concentrations and temperatures are shown in Equations (9) and (10).

*Final equation in terms of coded factors for gasometric:*

(9) $$Y=50.69+6.08X_{1}-2.49X_{2}-1.15X_{1}X_{2}$$

*Final equation in terms of coded factors for thermometric:*

(10) $$Y=48.31+6.23X_{1}-5.22X_{2}+0.57X_{1}X_{2}-0.22X_{1}^{2}+2.28X_{2}^{2}$$

By considering coefficient with significant terms, Equation (10) can be reduced to Equation (11) such that;

(11) $$Y=48.31+6.23X_{1}-5.22X_{2}+2.28X_{2}^{2}$$

However, a model modification may be considered if there are more redundant model terms than the significant terms.

### 3.3 Graphical representation of RSM and process optimization

**Figure 4:**
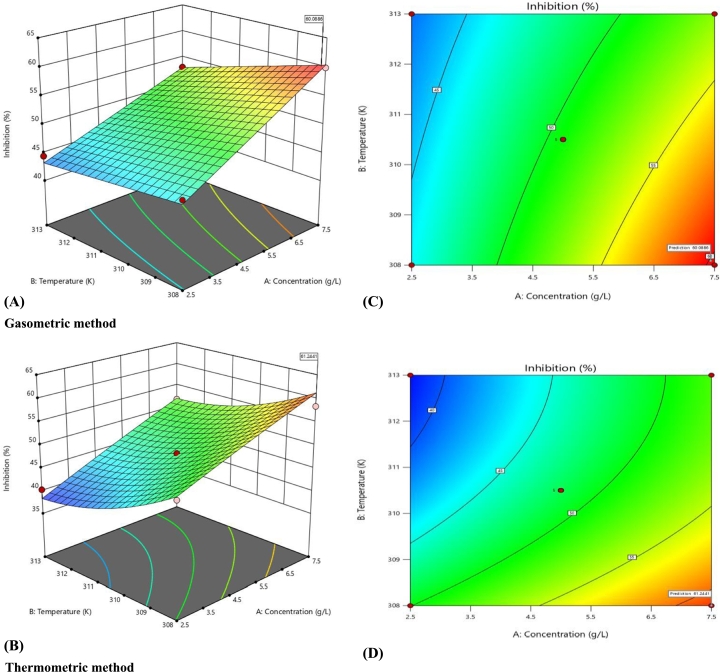
3-D Surface plots (A & B) and contour plots (C & D) showing interactive effects of concentration and temperature on mild steel corrosion inhibition efficiency of Banana peel extract obtained for gasometric and thermometric methods.

The response surface graphs (Figs. 4A & 4B) and contour plots (Figs. 4C & 4D) show the interactive effects of temperature and concentration on the efficiency of BPE as corrosion inhibitor. An elliptical contour shows an interactive effect, while a non-interactive effect is depicted by circular contours [32]. Hence, the elliptical contours observed in this study showed the interactive effects of concentration and temperature on the corrosion inhibition efficiency.

**Figure 5:**
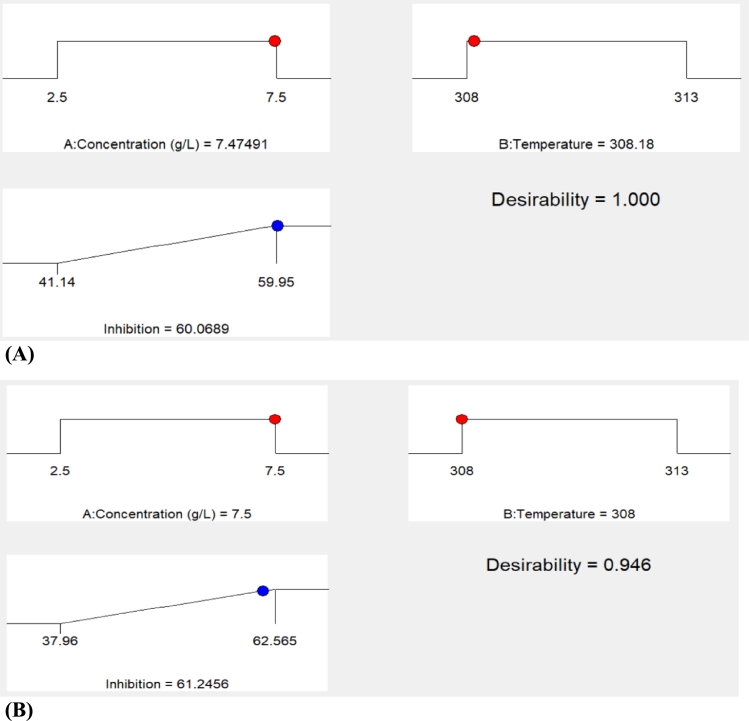
Numerical optimization of mild steel corrosion inhibition efficiency of Banana peel extract - gasometric method (A) and thermometric method (B).

Numerical optimization option of the DOE software was used to optimize corrosion inhibition efficiency. The input variables were set to maximise the efficiency of the extract to inhibit corrosion of mild steel. The software searches and generate desirability ramp, whereby the maximum desirability function corresponds to the optimum point. Thus, the highest corrosion inhibition efficiency of 60.69% was obtained at temperature 308.08 K and HCl concentration of 7.44 g/L for gasometric method (Fig. 5A). Meanwhile, for thermometric method, the highest inhibition efficiency of 61.25% was achieved at 308 K and concentration of 7.50 g/L (Fig. 5B).

### 3.4 Artificial neural network modeling

The result obtained from training the ANN with the Levenberg – Marquardt training model, at optimum neuron number while adopting the learning rule as momentum for training the experimental yield, gave a high R^2^ and low MSE value, indicating a better training and prediction by the ANN model. The optimum number of neurons for both gasometric and thermometric methods is 7, while the MSE values are 2.2788 and 2.7306, and R^2^ are 96.205 and 98.86, respectively (Table 7). Since the average absolute deviation (AAD) measures the variability of data set from the mean value, a higher AAD for thermometric method, shown in Table 7, indicates a higher statistical dispersion of the experimental data than those obtained with gasometric method. Comparing the performance models of RSM and ANN for both methods shown in Table 8; for gasometric method, R^2^ is 98.93 for RSM and 96.21 for ANN, while for thermometric method, R^2^ was obtained as 95.78 and 98.86 for RSM and ANN, respectively. Furthermore, the highest R square and lowest MSE value, obtained for 7 neurons (Table 8), showed the optimum system efficiency.

**Table 7:** Comparison of Response Surface Methodology (RSM) and Artificial Neural Network (ANN) performance models for gasometric and thermometric methods.

S/N	Conc. (g/L)	T (K)	EXP	R.S.M	ANN	EXP	RSM	ANN
			Gasometric method			Thermometric method		
1	7.5	303	64.14	67.671	64.140	62.4	88.788	62.400
2	5.0	313	51.11	48.202	51.110	41.93	45.372	41.930
3	1.0	303	44.40	42.922	44.400	41.58	76.690	41.580
4	10.0	313	61.75	58.060	61.750	59.85	58.077	59.850
5	2.5	308	49.53	45.953	49.530	49.25	49.928	49.250
6	7.5	318	49.25	45.860	49.250	44.98	60.892	44.980
7	7.5	313	53.14	53.130	53.140	51.73	51.948	51.730
8	2.5	318	40.28	40.593	40.280	33.94	45.013	33.940
9	5.0	308	56.31	53.177	56.310	54.13	55.811	54.130
10	2.5	313	44.43	43.273	44.430	40.31	38.349	40.310


Coefficient of determination (R^2^) and mean square error (MSE)				
	R.S.M	A.N.N	R.S.M	A.N.N

R^2^	98.93	96.205	95.78	98.86
MSE	15.67	2.2788	8.05	2.7306
AAD (Average Absolute Deviation)	3.7923		5.0828

**Table 8:** MSE and R square value for gasometric and thermometric methods.

Number of neurons	Mean square error (MSE)	R square	Mean square error (MSE)	R square
	Gasometric method		Thermometric method	
7	2.2788	0.96205	2.7306	0.98863
9	13.0162	0.95849	8.7276	0.95208
10	82.6328	0.9351	14.5711	0.96827
12	11.7601	0.9584	22.9150	0.94706
13	82.7852	0.7685	19.8936	0.97066
15	688.6764	0.7033	36.3375	0.82886
20	313.672	0.74576	72.4431	0.79266
25	49.1201	0.91158	160.694	0.91729
30	119.7959	0.616597	61.9347	0.95213

**Figure 6:**
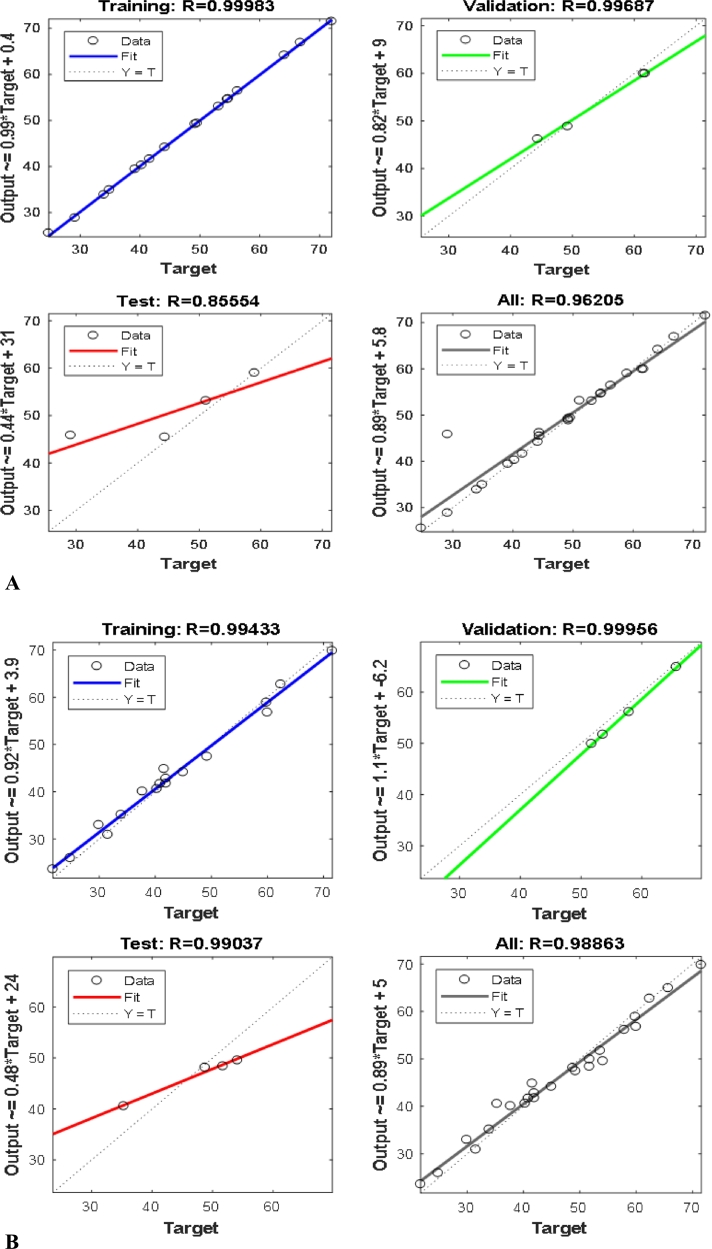
ANN regression plot for gasometric method (A) and for thermometric method (B).

The MSE and R^2^ value increases and decreases at different number of neuron, due to the simulation and adaptation of the number of neuron and how better it can predict the model. Significant R^2^ values of 0.9621 and 0.9886 were obtained for gasometric method and thermometric method, respectively, which implies an adequate prediction of the output layer because the closer the value of R^2^ to 1.0, the better and more significant the model. This correlation was further depicted by regression analysis shown in Figs. 6A and 6B.

**Figure 7:**
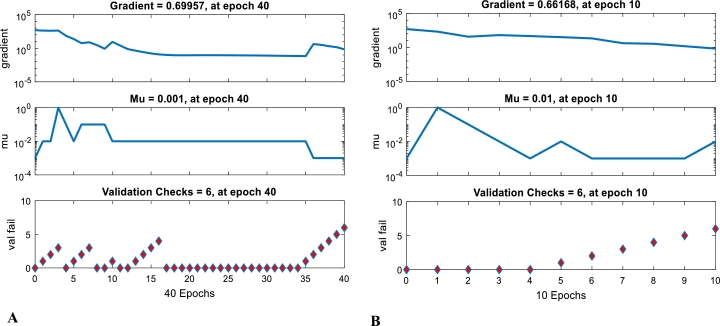
Artificial Neural Network training plots for gasometric method (A) and thermometric method (B).

The performance plots shown in Figs. 7 (A) and 7 (B) indicate the level of tolerance and failure of the training at the optimum number of neuron. These plots of gradient, mu (momentum) and Val (validity) fail versus epoch show that the training algorithm has acceptable training validity based on the input matrix. Hence, epoch numbers 40 and 10 were obtained for gasometric and thermometric methods, respectively, at optimum training of 7 neurons. The effectiveness of ANN to train smaller dataset has also been reported by Santhosh, et al. [50]

**Figure 8:**
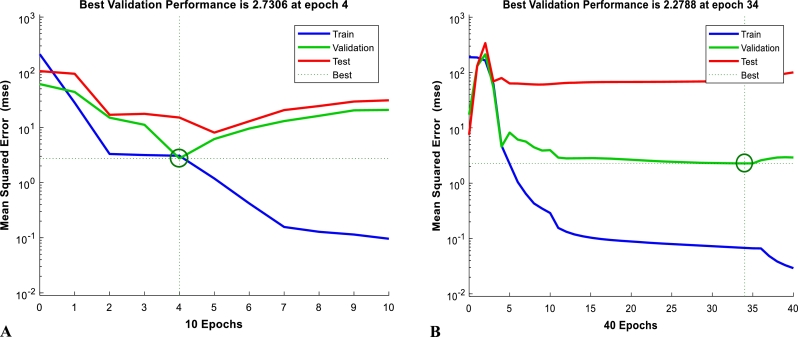
Artificial Neural Network performance plots at optimum neuron for thermometric method (A) and gasometric method (B) during optimization of mild steel corrosion inhibition.

Figs. 8 (A) and 8 (B) represent MSE profiles for thermometric method and gasometric method, respectively. The various MSE values, based on the ANN training, for training, validation and test are indicated with the blue, green and red, respectively. MSE actually reveals the extent of correlation of predicted data to experimental data by computing the deviation of the predicted data from the regression line (i.e. errors) and squaring them. The smaller the MSE, therefore, the closer it is to locating the line of best fit. Thus, the least MSE in this study was found to be 2.7308 at an epoch of 4 for thermometric method and 2.2788 at an epoch of 34 for gasometric method, both at the optimum neuron number.

## 4 Conclusion

The efficiency of banana peel extracts (BPE) as mild steel corrosion inhibitor in HCl was modelled and optimized using response surface methodology (RSM) and artificial neural network (ANN) algorithm. Also, the interactive effects of temperature and inhibitor concentration on inhibition efficiency was investigated, and corrosion rates assessed by hydrogen gas evolution. The evolution of hydrogen gas decreases with increasing concentration of BPE, while it increases with time for the various concentrations up till 8 min before attaining constant values.

The statistical analysis of the independent variables by ANOVA and response surface graphs showed an interactive effect on the corrosion inhibition efficiency; however, the effect of concentration was more significant. By numerical optimization option of RSM, the optimum corrosion inhibition efficiency of 61.25% was obtained at 308 K and HCl concentration of 7.47 g/L for thermometric method, while an optimum of 60.09% was obtained at 308.08 K and HCl concentration of 7.44 g/L for gasometric method.

Optimization of inhibition parameters with ANN reveals MSE to be 2.28 and 2.73 for gasometric and thermometric methods, respectively, while an optimum number of neurons was 7 for both methods. Comparing the performance models of RSM and ANN for both methods, RSM showed to be a better optimization tool with the gasometric method, while ANN gave a higher $R^{2}$ values and lower error values with the thermometric method. The possible reasons for the good prediction ability of ANN, even for small data set, may be due to its ability to adapt, learn and train dataset at reasonable learning rate of 0.1 as offered in the ANN simulation of the dataset. Also, within the exceptional features of ANN is the ability to detect all possible interactions between the predicting or independent variables. Overall, both RSM and ANN are robust tools for process modeling and optimization, as they reveal significantly $R^{2}$ values of over 90% for both methods.

## Declarations

### Author contribution statement

Author contribution statement: Olusola S. Amodu: Conceived and designed the experiments; Performed the experiments; Wrote the paper. Moradeyo Odunlami: Contributed reagents, materials, analysis tools or data. Joseph Akintola: Conceived and designed the experiments; Analyzed and interpreted the data. Olushola Ayanda: Performed the experiments; Wrote the paper. Tunde Ojumu: Contributed reagents, materials, analysis tools or data; Wrote the paper.

### Funding statement

This research did not receive any specific grant from funding agencies in the public, commercial, or not-for-profit sectors.

### Data availability statement

Data will be made available on request.

### Declaration of interests statement

The authors declare no conflict of interest.

### Additional information

No additional information is available for this paper.
